# A national study of substance use: Demonstrated use of recommendations for best practice online data collection

**DOI:** 10.1371/journal.pone.0336612

**Published:** 2025-11-10

**Authors:** Andrew P. Bontemps, Bailey E. Pridgen, William P. Wagner, Karen L. Cropsey

**Affiliations:** 1 Department of Psychiatry, University of Colorado Anschutz, Aurora, Colorado, United States of America; 2 Department of Psychiatry and Behavioral Neurobiology, University of Alabama at Birmingham, Birmingham, Alabama, United States of America; NYU Grossman School of Medicine: New York University School of Medicine, UNITED STATES OF AMERICA

## Abstract

**Objectives:**

Since the COVID-19 pandemic, more researchers have used to online data collection to recruit participants to research studies. However, one perceived limitation of online data collection is a belief that it results in lower quality data due to the introduction of bots or misrepresentation by participants to qualify for study compensation. The current study demonstrates that following recommendations for online data collection results in quick collection of a high-quality, diverse, multi-state sample.

**Methods:**

The current study followed recommendations for best practice, advertising on social media sites combined with investigator-implemented (e.g., splash page, attention checks, use of physical payment) and built-in Qualtrics tools (e.g., IP tracking, CAPTCHA) to collect data from participants who use substances from 15 states within the United States examining cannabis use and perceptions of harm reduction interventions (HRIs).

**Results:**

Before cleaning, 3,642 participants completed the screener across 172 days of survey up-time. After cleaning, the final sample included 639 responses in the final cannabis survey, and 1,137 responses in the final HRI survey including 264 participants completing both surveys. The study yielded approximately 8.8 cleaned surveys per day and a usable data rate of 60.3% for participants who completed the cannabis survey only, 72.4% for participants who completed the HRI survey only, and 72.6% for participants who completed both.

**Conclusions:**

While every method of data collection has strengths and weaknesses, when implemented using appropriate tools to prevent completion of surveys by non-valid participants, internet-based data collection methods can provide researchers with relatively low-cost, high-quality samples.

## Introduction

While online data collection has been utilized by researchers since the proliferation of the internet, the COVID-19 pandemic necessitated that many in-person studies significantly change to methods allowing for remote data collection for part or all of their study [1,2]. Platforms like Amazon Mechanical Turk (MTurk) and Prolific Academic (Prolific) and research panels provided by companies like Qualtrics and Ipsos allow researchers to access large populations and to collect data quickly and economically, especially compared to in-person data collection [2–6]. However, while these platforms increase researcher and participant ease-of-use, there is controversy over the data quality achieved from the platforms [7–10] as well as demographic differences from online panels compared to the U.S. population [11,12].

Although shifting to remote methods allows for the collection of national and international datasets, it leaves room for individuals to illegitimately claim monetary research incentives by speeding through research or through the use of bots to complete surveys [10,13]. Past meta-research investigating internet-based research has shown that data collected from platforms like MTurk may include more data quality issues compared to in-person data collection and may have duplicate IP addresses (i.e., indicating one individual attempting to complete the survey multiple times) [13–15]. Research has suggested that CAPTCHA may be useful in bot detection, but a CAPTCHA alone may not be sufficient for reducing bots’ ability to complete surveys undetected [15,16]. Naturally, these attempts to take advantage of research incentives have been met with consternation within the research community [4,7,10,15,17], prompting the need for standards for internet-based research.

In response, Newman and colleagues recently published recommendations for online survey recruitment to both improve the validity of responses and achieve broader representation in the sample [4]. Their recommendations include using platforms with diverse participant pools; use of platforms with non-native users (i.e., limiting access from those who complete lots of online surveys); incorporating attention checks; utilizing built-in tools to flag participants with suspicious or duplicate IP addresses; developing appropriate compensation (i.e., by not rewarding too much or too little); and increasing transparency of the study goals, benefits, and potential risks [4]. For social media recruitment specifically, King and colleagues recommended using an intermediary “splash page” between social media advertisements and the study itself, putting an upper limit on the amount of time participants can take to complete the study, and changing advertisements to elicit higher rates of clicks [3]. The splash page, also referred to as a home page or landing page in the literature, provides additional information about the study in plain language and gives participants additional opportunities to opt-in or -out of the survey before viewing the consent document [3,18]. Similarly, Hardesty and colleagues have published a recent account of their initial problems and list of recommendations for best practices when conducting social media-based internet research. Specifically, they recommend a process for determining valid cases changed while data were being collected, as the researchers noted an influx of fraudulent responses. Thus, the current study benefited from more recent recommendations for online data collection (e.g., CAPTCHA, recording IP address, attention checks), as these validation strategies were able to be integrated from the beginning of data collection [15].

### Substance use research in the United States

Large public datasets of substance use within the United States, such as Monitoring the Future (MTF) [19,20] and the National Survey on Drug Use and Health (NSDUH) [21], are publicly available and widely published to provide epidemiological estimates of use, but the scope of the questions asked necessarily limits substance use researchers. For example, the NSDUH asks people who used “marijuana/cannabis” in their lifetime to report the approximate date of first use, time since last use, approximate days of use in the past year, approximate use frequency in the past month, and methods of consumption. While thorough, the series of questions do not include all salient aspects of cannabis use. For example, the types of cannabis consumed (e.g., Δ-9 THC, Δ-8 THC, HHC) may vary dramatically depending on state legal status of cannabis, and motivations for cannabis use are integral for developing interventions for problematic use. Therefore, substance use researchers may want to collect mid- to large-scale survey data on specialized topics, supplementing information provided from national epidemiological studies. In order to ensure the survey data are reliable, additional studies are needed to investigate the methods by which internet-based research in the social sciences is conducted, especially in more sensitive topic areas like substance use.

Additionally, there have been very few studies investigating the method by which participants who use substances can be recruited from internet-based sources. For instance, while some more recent papers make note of the benefits of using internet-based tools to maintain study engagement [22], how to design effective surveys for measurement of various substances [23], the types of data that can be collected online at the population level [24], and the need to pivot to internet-based research to support clinical trials [25], few provide a guide for how to conduct internet-based research for SU. Of those that do, McCabe and colleagues [26] found that participants who completed a “Web survey” were more likely to respond to and match national data compared to a U.S. mail survey and described how to design and distribute a survey on the web. More recently, Bauermeister and colleagues [27] described a method by which initial survey participants were found through social-media-based advertising and asked to give contact information. The researchers then used that information to call these participants (i.e., “seed participants”) individually to verify their identity and answer questions about participation. Further participants were contacted via email by researchers through snowball recruitment from the initially verified seed participants as a method of ensuring that additional participants were also likely not be bots [27]. They also noted daily manual checks to ensure data quality and disallowing fraudulent participants to forward email recruitments to additional participants [27]. The current study will seek to provide further updates to the previously suggested methods for internet-based SU research, combining them with recommendations for internet-based research more broadly.

Thus, by following a combination of the recommended research methods outlined above [3,4,15,16], the current study aims to collect one of the largest independent samples of individuals who use substances and to serve as another model for future researchers [15]. The researchers utilized online social media-based recruitment methods to collect data on substance use (SU) and psychosocial factors of individuals from 15 U.S. states, and the current paper describes the methods used to maximize the likelihood of collecting high quality data.

## Methods

The current study contained two surveys investigating substance use in the United States: one survey asked participants about various aspects of cannabis use, and the other survey asked participants their experience and perceptions of harm reduction interventions (HRIs) related to substance use. Depending on their eligibility, participants could have completed either one or both of the surveys. If participants qualified for both surveys, they needed to complete the cannabis survey before starting the HRI survey. Before either survey, all participants completed a screener that included demographic information, screening questions, and other questionnaires. S1 Table provides more information on the questions included in each survey.

### Participants

Participants were eligible for the cannabis survey if they reported past-90 day use of “Cannabis (Marijuana, Pot, Weed, THC, CBD, etc.).” Participants were eligible for the HRI survey if they reported lifetime use of opioids (i.e., heroin, fentanyl, or illicit prescription opioid use) or stimulants (i.e., methamphetamine, cocaine, MDMA, or illicit prescription stimulant use). Participants were ineligible for both surveys if they did not live in any of the 15 U.S. states surveyed (see Table 1 for full list of included states). The cannabis survey aimed to recruit 800 participants, and the HRI study aimed to recruit 1,200 participants. All study methods were approved by the University of Alabama at Birmingham IRB (IRB-3000010066).

**Table 1:** Participant Characteristics.

	Number Completed Cleaned Screener	Number Completed Cleaned Cannabis	Number Completed Cleaned HRI	Number Completed Both Studies
**Total *N***	2747	639	1137	264
	M (SD)			
**Age**	45.0 (10.8)	43.6 (10.5)	45.4 (10.5)	44.39 (10.13)
	N (% of total)			
**Participant Sex**				
Male	784 (28.5)	177 (27.7)	332 (29.2)	65 (24.6)
Female	1910 (69.5)	441 (69.0)	785 (69.0)	189 (71.6)
Non-binary/Genderfluid	29 (1.1)	9 (1.4)	12 (1.1)	6 (2.3)
Transgender Male	14 (0.5)	5 (0.8)	5 (0.4)	1 (0.4)
Transgender Female	7 (0.3)	4 (0.6)	1 (0.1)	1 (0.4)
Other	3 (0.1)	3 (0.5)	2 (0.2)	2 (0.8)
**Race**				
African-American/Black	360 (13.1)	80 (12.5)	138 (12.1)	25 (9.5)
Latino	143 (5.2)	30 (4.7)	57 (5.0)	11 (4.2)
Asian	25 (0.9)	5 (0.8)	18 (1.6)	4 (1.5)
Native American/Alaska Native	28 (1.0)	5 (0.8)	11 (1.0)	2 (0.8)
Native Hawaiian/Pacific Islander	1 (0.0)	0 (0)	0 (0)	0 (0)
Caucasian/White	1931 (70.3)	458 (71.7)	806 (70.9)	205 (77.7)
Arabic/Middle Eastern	5 (0.2)	2 (0.3)	2 (0.2)	1 (0.4)
Indian	5 (0.2)	2 (0.3)	1 (0.1)	1 (0.4)
Multiracial	234 (8.5)	54 (8.5)	99 (8.7)	14 (5.3)
Other Race	15 (0.5)	3 (0.5)	5 (0.4)	1 (0.4)
**Ethnicity**				
Hispanic	331 (12.0)	58 (9.1)	139 (12.2)	22 (8.3)
Non-Hispanic	2416 (88.0)	581 (90.9)	998 (87.8)	242 (91.7)
**State of Residence**				
California	507 (18.5)	62 (9.7)	206 (18.1)	22 (8.3)
Georgia	181 (6.6)	58 (9.1)	80 (7.0)	24 (9.1)
Idaho	29 (1.1)	3 (0.5)	14 (1.2)	0 (0)
Illinois	198 (7.2)	46 (7.2)	77 (6.8)	18 (6.8)
Kentucky	230 (8.4)	50 (7.8)	96 (8.4)	18 (6.8)
Mississippi	82 (3.0)	29 (4.5)	29 (2.6)	14 (5.3)
New Hampshire	11 (0.4)	6 (0.9)	4 (0.4)	3 (1.1)
New Mexico	56 (2.0)	12 (1.9)	15 (1.3)	2 (0.8)
New York	235 (8.6)	52 (8.1)	96 (8.4)	20 (7.6)
Ohio	292 (10.6)	93 (14.6)	138 (12.1)	41 (15.5)
Oklahoma	160 (5.8)	56 (8.8)	67 (5.9)	23 (8.7)
Texas	451 (16.4)	94 (14.7)	186 (16.4)	43 (16.3)
Utah	88 (3.2)	24 (3.8)	39 (3.4)	11 (4.2)
Virginia	115 (4.2)	29 (4.5)	56 (4.9)	17 (6.4)
Wisconsin	109 (4.0)	25 (3.9)	34 (3.0)	8 (3.0)
Missing	3 (0.1)	–	–	–
**Urban Zone**				
Urban	1358 (49.4)	285 (44.6)	537 (47.2)	105 (39.8)
Suburban	916 (33.3)	228 (35.7)	396 (34.8)	97 (36.7)
Rural	471 (17.1)	125 (19.6)	202 (17.8)	61 (23.1)
Missing	2 (0.1)	1 (0.2)	2 (0.2)	1 (0.4)

Note: Completed Cleaned Cannabis and Completed Cleaned HRI columns include participants who completed both studies.

Participants were recruited through advertisements on Meta-based social media sites (i.e., Facebook and Instagram) in the 15 states. The states were selected to represent states that had legalized recreational cannabis use for adults, legalized medical cannabis use for adults, or maintained non-legal status of cannabis at the time that data collection began (i.e., August 2023). Additionally, the states were selected as they represented, as best as possible, the geographical regions of the United States (i.e., Northeast, Southeast, Midwest, Southwest, West) while considering cannabis legalization status (see Table 2 for cannabis and harm reduction policies at time of recruitment). The authors hoped to collect data that accurately reflected a wide range of regions and social experiences within the United States.

**Table 2:** Legal status of cannabis and HRI method.

State	Cannabis Legalization Status	FTS^1^ Permitted	XTS^2^ Permitted	SSP^3^ Authorized	SSP^3^ Open	SIS^4^ Open
California	Medical & Recreational	Y	Y	Y	Y	Y
Georgia	No Legalization	Y	N	Y	Y	N
Idaho	No Legalization	N	N	Y	Y	N
Illinois	Medical & Recreational	Y*	Y*	Y	Y	N
Kentucky	No Legalization	Y	N	Y	Y	N
Mississippi	Medical only	Y	N	N	N	N
New Hampshire	Medical only	Y*	Y*	Y	Y	N
New Mexico	Medical & Recreational	Y	Y	Y	Y	N
New York	Medical & Recreational	Y	Y	Y	Y	Y
Ohio	Medical only	Y	N	Y	Y	N
Oklahoma	Medical only	Y	N	Y	Y	N
Texas	No Legalization	N	N	N	Y	N
Utah	Medical only	Y	Y	Y	Y	N
Virginia	Medical & Recreational	Y	Y	Y	Y	N
Wisconsin	No Legalization	Y	N	N	Y	N

Note: The legalization status in the table represents status during the study period of August 2023 – February 2024. *Indicates that legalization was passed during the recruitment window

^1^FTS: fentanyl test strips.

^2^XTS: xylazine test strips.

^3^SSP: syringe service programs.

^4^SIS: supervised injection sites.

### Study design

The current study’s surveys were built in Qualtrics, and participants accessed them via a public link. The researchers initially considered data collection through online recruitment platforms (e.g., MTurk, Prolific) and catered research panels (e.g., Ipsos, Qualtrics’ online samples), but these methods were not feasible because of institutional restrictions and practicality of data collection. The study was conducted in the United States at an institution that limited the researcher’s ability to develop contracts with Amazon or institutions based outside the U.S. due to institutional policies. Thus, the study could not be conducted using MTurk or Prolific, respectively. Additionally, while services like Ipsos and Qualtrics’ online samples allow access to data panels, many do not allow research questions that ask about illegal activities (e.g., illicit SU). Thus, the current project was conducted with a public link that was advertised on social media with the assistance of an external marketing firm.

Advertisements were collaboratively created for the project by the marketing firm and research team and approved for use by the marketing department of the authors’ institution and IRB (see S1–S3 Figs for example of two advertisements and the splash page, respectively). The marketing team managed the ad campaign by purchasing ads on the selected websites; restricting ads to users in the selected states; and adjusting recruitment needs (e.g., targeting ads toward individuals who had lower incomes, balancing an initial influx of high-income participants).

Advertisements gave a brief introduction to the study topic and provided a link to the study landing page (e.g., “Join an online UAB research survey of SU in adults across the U.S. Receive compensation & help make a difference.”). Advertisements on social media started on 08/25/2023 and ran through 02/13/2024 with two brief pauses between 09/01/2023–09/06/2023 and 09/14/2023–10/09/2023 due to an error in the Qualtrics survey that caused a problem collecting date of birth. If potential participants clicked on an ad, they were directed to a landing page that included a link to the start of the study and a description of the study and requirements for participation broadly (e.g., “Through a national online survey we hope to understand what substances people are using, their motivations for using, and to gather feedback on available treatments.”).

If a potential participant clicked the link on the landing page, they were directed to the consent document, which was included as the first page of the Qualtrics survey. Once digital consent was obtained, participants were directed to the screener, followed by the survey(s) for which they qualified. When participants completed each survey (i.e., cannabis or HRI) they were informed that they had completed a study and given the opportunity to opt-in or opt-out of payment. Compensation was provided via prepaid debit cards operated through the Greenphire ClinCard system. To receive compensation, participants were required to provide their name, date of birth, and mailing address. Study authors obtained a waiver to not collect participants’ social security number.

Once participants completed all surveys for which they had qualified and opted-in or -out of compensation, they were thanked for participating and shown a page that provided researcher contact information and links to find mental health support broadly or SU treatment in their area.

### Data quality assurance

To ensure data quality, the authors followed recommendations from previously published best practices for online data collection [3,4]. The current study included a splash page giving potential participants additional information about the study after clicking an ad. Practically, the additional information should allow potential human participants a more informed decision about participation [3], though it includes less information than the informed consent document. The current study also utilized built-in Qualtrics tools intended to prevent multiple attempts at taking the study and to identify potential bots (i.e., CAPTCHA). Attention checks (i.e., two questions) were included in the screener and asked participants to select a specific response to indicate full attention. Responses to all questions were required to advance through the study to reduce the potential of “speeding” (i.e., racing through questions) and to eliminate skipping. All participant data were removed if they failed the CAPTCHA (i.e., scored 0.5 or lower) [28] or failed either attention check in the screener. Within the cannabis study, additional fake types of “cannabis” (i.e., Δ-15 THC, HSBC) were included in the list of cannabis types that participants could endorse for past 90-day use. If a participant selected a fake type of cannabis in the cannabis survey, then their cannabis survey data were removed as it was assumed that they had insufficient knowledge of their cannabis intake. If a response had discrepant names (i.e., different names in the compensation data for the two surveys) or discrepant address information (e.g., reported living in a different state from their mailing address), then their data were removed. If multiple responses had duplicate IP addresses, names, or addresses, the response was then reviewed and, if they were determined to be from the same person, only the first response was kept. When checking for duplicates, each response was compared to every other response in the raw dataset (i.e., before any removals based on CAPTCHA, attention checks, or survey completion).

### Measures

Measures for the current study were distributed across the screening questionnaire, the cannabis survey, and the HRI survey, summarized in S1 Table. The screener contained a total of 10 questionnaires representing 66–77 total questions, the cannabis survey contained 10 questionnaires representing 175–733 questions (dependent on how many types of cannabis were used); and the HRI survey contained 6 questionnaires representing 91 questions.

## Results

### Participants

Social media advertisements for the study were clicked 19,785 times. Of those, 6,213 individuals clicked through to the study from the splash page, and 5,128 individuals completed the consent document, representing an advertisement conversion rate of 25.9%. Of the individuals that completed the consent, 20 declined participation, leaving 5,108 consenting participants. Of the participants who consented, 1,050 started but did not complete the screener, leaving 3,764 (73.7%) participants who completed the screener. Of the participants who completed the screener, 632 participants completed only the cannabis survey, 1,143 completed only the HRI survey, and 427 completed both surveys for a total completion rate of any study of 2,202 participants before cleaning (i.e., 60.5% of those who completed the screener).

**Fig 1:**
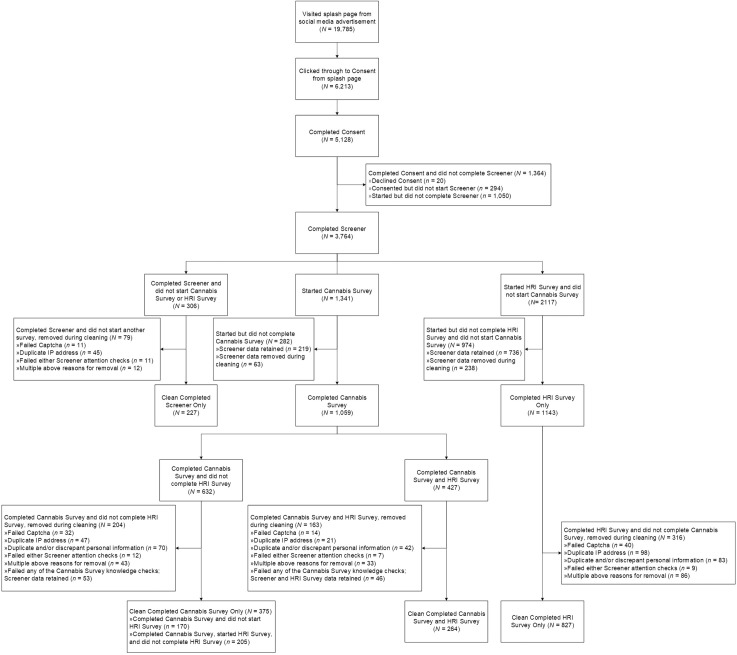
Consort diagram of participants.

Of the 632 participants who completed only the cannabis survey, 257 (40.7%) were removed during the cleaning process leaving 375 participants who completed only the cannabis survey. The median time to complete the screener and cannabis survey was 40 minutes (IQR = 32 minutes, 60 minutes). Of the 1,143 participants who completed only the HRI survey, 316 (27.6%) were removed during cleaning, leaving 827 participants who completed only the HRI survey. The median time to complete the screener and HRI survey was 32 minutes (IQR = 25 minutes, 46 minutes). Lastly, of the 427 that completed both surveys, 117 (27.4%) were removed, leaving 264 participants who had completed both surveys. The median time to complete the screener, cannabis survey, and HRI survey was 61 minutes (IQR = 44 minutes, 95 minutes). An additional 46 participants who completed both surveys had their data from the cannabis survey removed due to failed cannabis knowledge checks. See Fig 1 and Table 3 for more details on why responses were removed during the cleaning process.

**Table 3:** Number of incomplete and complete study participants by study.

	Screener		Cannabis Only		HRI Only		Qualified for Both Studies	
	Incomplete	Finished	Incomplete	Finished	Incomplete	Finished	Cannabis complete; HRI incomplete	Finished
Total	1050	3764	282	281	613	1143	351	427
Removed due to Duplicate IP	201	378	32	28	135	98	19	21
Removed due to Duplicate or Discrepant Personal Information (Address/Name)	0	195	0	28	0	83	42	42
Removed due to Failed CAPTCHA	50	185	19	16	69	40	16	14
Removed due to failed screening survey attention checks	21	65	8	7	18	9	5	7
Multiple reasons for removal	38	194	4	5	6	86	38	33
Not Removed for any of those reasons	740	2747	219	197	736	827	231	310
Removed cannabis survey responses because failed one or more cannabis survey attention checks	–	–	23	27	–	–	26	46
Total after cleaning		2747		170		824	205	264
			Cannabis		HRI			
Survey Completions After Cleaning				639		1137		

Therefore, 639 participants were included in the final cannabis survey sample and 1,137 in the final HRI survey sample, giving a usable data rate of 60.3% for participants who completed the cannabis survey only, 72.4% for participants who completed the HRI survey only, and 72.6% for participants who completed both surveys. Thus, after cleaning, the cannabis survey was at 79.9% of the original recruitment goal, and the HRI survey was at 94.8% of the original recruitment goal. Participant demographics of participants who were removed due to cleaning procedures were similar to the demographics who completed the surveys and were maintained in the final sample (See S2 Table). Finally, as the study ran for approximately 172 days, the current study collected an average of 8.8 cleaned participant surveys per day, indicating relatively quick data collection.

### Study cost

As noted in previous studies, the cost of the current study was divided between two components: advertisements and participant compensation [18]. Due to institutional requirements, the current study was conducted with an external marketing team who developed advertisements collaboratively with the study team. The marketing team was paid $17,900 for development and management of the social media advertising campaign. In addition, participants were credited $10 per survey completed, for a total of $20 if they completed both surveys. Participants were not compensated if they only completed the screener, and participants who completed the cannabis and/or HRI surveys did not receive extra compensation for completing the screener. Notably, declining payment was very rare. Only two participants in the cleaned cannabis dataset declined payment, seven participants in the cleaned HRI data set declined payment, and another five participants answered every question on the HRI survey but did not respond to the payment question. The study was charged $1.50 for each pre-paid debit card mailed to participants and about $0.52 to mail each card. The researchers strove to provide payments to each participant within eight weeks of when they completed the survey. Data were downloaded monthly and initial validity checks were conducted. In order to expedite the payment process, it was accepted that some participants who were not included in the final dataset would receive payment, A total of $17,200 in participant funds were distributed and $2,847.19 in charges were accrued for sending debit cards. Thus, the total costs of conducting this study (excluding staff time to mail the cards and clean/review the data) was approximately $37,947.19. This amounts to an approximate total of $17.23 per completed survey before cleaning and $25.88 per cleaned survey. Cost per participant is higher than previously reported costs (e.g., ranging from a median cost of $0.40 to $4.33 per participant) largely due to the outsourcing of the advertising campaign and compensation commensurate with what participants would be compensated for an in-person questionnaire-based survey [18].

### Participant characteristics

Participants who completed the cannabis survey (N = 639) had an average age of 43.6 (*SD* = 10.5). More female participants (69.1%) and White participants (71.8% White; 90.9% Non-Hispanic) than would be expected from the U.S. population completed the cannabis survey. Similarly, participants who completed the HRI survey (*N* = 1137) had an average age of 45.4 (*SD* = 10.5) and included slightly more female (69.0% female) and White participants (70.9% White; 87.8% Non-Hispanic) than would be expected. Participants who completed both (*N* = 264) had an average age of 44.4 (*SD* = 10.1) and, like the individual studies, included more female (71.6%) and White participants (77.7% White; 91.7% Non-Hispanic). See Table 1 for full details of participant demographics by survey.

Finally, participants were recruited successfully from all states, but there was a wide range in overall response rates ranging from 10 participants (i.e., 0.6%) responding from New Hampshire to 280 (i.e., 15.7%) responding from Texas. Likewise, the current study successfully recruited from all types of residential zones including 822 (46.3%) from urban, 624 (35.1%) from suburban, and 327 (18.4%) from rural areas. See Table 1 for full details of participation by state and urban zones.

## Discussion

The present study contributes to the field of SU research in multiple ways. First, it serves as a practical example of how a large sample of data can be collected and then cleaned using easily replicated, internet-based data collection. In total, 19,785 individuals clicked on advertisements, 5,128 completed the consent document, 2,209 participants completed one or both studies, and 1,512 participants had clean data in one or both studies. Thus, while there was a clicked-advertisement to clean data rate of 7.6%, the clean data rate of individuals who completed any study was 68.4%, significantly more than previously reported data on participants recruited from MTurk [10]. With regard to bot detection, of the 5,844 participants who completed the screener and/or any of the studies (i.e., cannabis, HRI, or both), only 271 (4.6%) were removed due to failing the CAPTCHA and 544 (9.3%) were removed due to duplicate IP addresses. While CAPTCHA failure and duplicate IP addresses do not necessarily indicate completion of the survey by bots—in fact, duplicate IP addresses may indicate multiple individuals attempting to *validly* complete the survey from the same device (e.g., multiple members of a single household)—they can serve as a close proxy and show the necessity of taking steps to prevent bot-completed surveys. Lastly, concerns about the accuracy of data due to participants’ attention to detail were addressed through multiple attention checks, giving confidence in self-report information collected online.

Second, it should be noted that data collection was relatively fast. The current study was able to recruit approximately nine valid participants per active study day and reached full recruitment within about six months. This aligns with other studies that were completed using social media-based recruitment [15,29]. The current study also stands out in that it includes a breakdown of how much was paid to firms to advertise and run the study, as well as how much was paid out to participants in research incentives. While some prior researchers have noted study costs, the current researchers found that greater costs were needed per participant than in prior studies (i.e., $12.21 of advertising costs per cleaned survey compared to previously reported $0.40-$4.33) [18]. This cost is likely elevated compared to previous research as a result of institution-mandated use of a third-party marketing firm and may be reduced if research teams produce their own advertisements. The elevated cost may also be due to the current study’s focus on individuals who use illicit substances, mirroring prior research that found higher ad-spend was needed to reach traditionally hard-to-reach individuals [30]. However, the current study showed higher, but relatively similar, “cost per click” (i.e., amount of ad spend per time advertisements were clicked) to previous research (i.e., $0.90 in the current study compared to $0.66 in prior research) [30] and a total cost per cleaned participant similar to previous findings [31]. As stated by both Zindel [31] and others [18], transparency in total study price, price per click, and price per cleaned participant is necessary for future researchers trying to follow previous recommendations to understand the amount of advertising to purchase as well as to price study incentives correctly ensuring that participants are adequately incentivized, but not tempted to take the study repeatedly to gain multiple instances of study incentives [4].

Third, the current study showed that while the sample was older, more female, and more Non-Hispanic White than one would expect from the U.S. population [32,33], the sample also followed similar trends compared to previous studies investigating the demographic makeup of online survey sites. For example, a study investigating the differences between undergraduate, social media, and MTurk recruitment found that undergraduate recruitment overrepresented young participants and was slightly more female and White; social media recruitment strongly overrepresented young, White, and female participants; and MTurk strongly overrepresented young, Asian, and male participants [11]. Similarly, a 2019 study found that MTurk workers were significantly younger than community samples, overrepresented male, White, and Asian Americans, and underrepresented both Black and Hispanic Americans [12]. Another study found that individuals across MTurk and a commercial research panel had non-significant oversampling of female participants, but those recruited from the research panel were significantly older than those from MTurk [6]. Thus, while the current study showed differences in the sample compared to the U.S. population, it contributes to the body of literature showing the demographic makeup of recruitment methods. Future researchers can use a combination of screeners that ask participant demographic information with built-in quotas through a service like Qualtrics or REDCap, enabling better demographic control.

Finally, to our knowledge, the current study is one of the largest independent studies of SU across multiple states within the United States. Prior studies often focused on single states [34,35] or on multiple states or trends while using data taken from large national studies (e.g., the NSDUH or the Population Assessment of Tobacco and Health Study) [35–39]. These methods have notable benefits, not least of which is convenience in obtaining a sample that is nationally representative. However, single-state studies cannot investigate effects of different state laws, and secondary data analysis restricts researchers to the variables included in the original study. The current study demonstrates the possibility of conducting independent multi-state SU research using internet-based data collection techniques in a relatively short amount of time, and future research building on the guidelines in this study may further refine and reduce both cost and time needed to do this kind of wide-scoping research. To this end, the current study advertised only on social media websites that required an account and in states where recruitment was expected (i.e., in researcher-selected states). Additionally, the current study integrated a splash page to explain the study protocol and utilized both in-built (e.g., IP address monitoring, CAPTCHA) and study-design tools (e.g., screener, attention checks, card-based payment) to maximize participant attention and minimize bots completing the study undetected [28].

The above recommendations and more were recently implemented by Hardesty and colleagues [15] in a publication detailing their difficulties and ultimate successes in implementing large online methods for data collection. In their study, they detail how they developed a survey that was quickly flooded with fraudulent survey submissions and how they altered their study to mitigate risk in online data collection [15]. Their pre-survey recommendations for researchers include requesting broad leeway from IRBs to remove participants suspected of survey misuse. Within the survey, they recommend authenticating survey takers through recording cellphone numbers, IP addresses, and CAPTCHA scores, shortening the overall data collection period (i.e., the amount of time the survey is active) to limit individuals’ window of time to assess how to best “game” the survey, generating random URLs to stop individuals from “guessing” or retaking the survey, requiring photo or written submissions, and using warnings in the consent against survey misuse and consequences of misuse. After the survey is completed, they recommend authenticating names, date of birth, and mailing address, contacting participants to request verification of name and address (i.e., through the use of an ID card or bill) as needed if names are not initially authenticated, mailing survey incentives to physical addresses, and completing initial data checks before mailing incentives [15]. In the study, the authors noted that the mitigation strategies they recommend were easily implemented, scalable, and cost-effective [15].

Similar to Hardesty and colleagues’ study [15], the current study was able to easily implement mitigation strategies and did so from the beginning of data collection, a notable advantage over previous studies and largely due to following recommendations that came to light after the Hardesty study began [4,12,13,17]. However, it should also be noted that the field of internet-based data collection is ever-changing. For example, while King and colleagues [3] made many recommendations that still apply today, their paper stood at the relative beginning of using social media recruitment. As a result, their recommendation to “create a dedicated study web site” still stands as a time-tested recommendation [15], while their recommendation to use direct online payments is being replaced by a recommendation to mail physical incentives to deter people from repeating surveys, manually or with bots, to repeatedly qualify for research incentives [15]. Though the current study represents a synthesis of previous recommendations for internet-based research and can serve as a starting point for others trying to conduct similar research, the current protocol is not a comprehensive guide, and future researchers must continue to adapt to changes in the data collection landscape. Specifically, some recent studies have started to point out that bots powered by artificial intelligence may be even more skillful in completing internet-based research without detection and there are few current studies that specifically detail how researchers might be able to detect these AI-powered bots [40,41].

### Limitations and strengths

Though the present study made considerable efforts to utilize and expand upon best practices of online research, our methodology was limited in several ways. First, all data were self-reported by participants. A recent systematic review on the agreement between self-reported illicit SU and biological measures of use (e.g., blood, urine, hair. and saliva samples) found that consistency was high when collected for research purposes in which there are no negative consequences of disclosing SU [42]. Considering the meta-analytic findings and study measures taken to reduce the amount of personal information collected, there is a high likelihood that participants accurately reported SU.

Second, while the current study sought equal representation from all included states, it was not possible to achieve equal numbers of participants from some of the less-populated states (e.g., New Hampshire, Idaho; See Table 1). However, the researchers were able to adjust data collection to ensure collection of an overall sample that met recruitment goals, in spite of the limited responses from smaller states. Future studies may wish to increase participation in states with smaller populations by running studies for longer in those states or using multiple data collection methods (e.g., social media recruitment *and* the use of MTurk or Prolific).

Third, the study was limited to people who had internet access and were able to view social media ads through a Facebook or Instagram profile. When developing the recruitment strategy, it was apparent that data collection was going to be limited to persons with accounts on specific websites (e.g., Meta-based Facebook or Instagram, Prolific, MTurk, etc.) unless recruitment was conducted at a wide scale across multiple platforms (e.g., through Google advertising), at a high monetary cost. Thus, while platform-specificity is a limitation of the current research, this is also a limitation of most internet-based recruitment strategies.

Fourth, the current study found that the sample recruited from Meta-based sites were slightly older, more female, and more non-Hispanic White than would be expected [32]. However, the demographic makeup of persons screened in the study was similar to, if not slightly more, reflective of the U.S. population compared to other studies utilizing internet-based data collection [11,12], showing the feasibility of collecting diverse samples using social media-based recruitment methods. As a strength, the study sample also included a large number of persons living in rural and suburban areas. Prior studies examining SU have been largely conducted in urban populations [43–46]. The current study successfully reached a population underutilized in SU research, making a significant contribution to SU research.

The current study also used pre-paid debit cards sent via mail as a payment method for participants. Debit cards served as both a strength and limitation for the study. Because participants were required to give an address within the United States to be compensated for study completion, participant addresses could be used as a guard against attempts to illegitimately complete the study or give false information (e.g., indicate that they lived in one state on the screener and send payment to another state). However, participants may have been disincentivized to report potentially illegal activities (i.e., SU) knowing they would need to divulge personal information to receive compensation, as well as wait up to eight weeks for that compensation. Further, sending cards through the mail is not feasible for people who are unstably housed or who change residences frequently, a significant problem among persons with SU. Researchers worked with their institution’s IRB to reduce participant burden of information that was required to receive compensation (i.e., social security number), and future researchers may wish to consider the cost-benefit of collecting participant information for payment compared to utilizing built-in participant payment systems.

Finally, the authors used what may be stricter-than-necessary cutoffs in cleaning (e.g., excluding participant data when they failed even one attention check, had a previously recorded IP address, or had any discrepant personal information). The decision to strictly exclude data was made to demonstrate that, even when removing unreliable data with the strictest possible cutoffs, a large amount of retained responses from a broad sample could still be quickly collected. Past research has specifically stressed the importance of attention and attention checks to ensure the collection of high-quality data from valid participants in online research and the importance of using multiple tools to ensure that non-valid participants (e.g., those trying to take the same study multiple times, those trying to “speed” through the study to receive compensation quickly, bots) are removed from the final data set [4,3,18]. At the same time, it is important to recognize that using stricter cutoffs may lead to the removal of some valid data [8,10,18]. For example, using the current protocol, if two individuals living at the same address completed the study on the same device, their data may be removed as the data would have the same IP address and same home address. Additionally, while the current study did not use cut off times (e.g., cutting participants who took too long to complete the survey) the use of cut off times may inadvertently cut participants with lower technological literacy, participants with lower English literacy, or older adults. In turn, by implementing cutoffs researchers may introduce bias into the sample in their attempts to collect more valid data. Thus, it is important that researchers consider the potential trade-offs when selecting criteria for removal of data and participant payment to maximize the number of valid responses while also ensuring high-quality data. Researchers may also choose to use quotas to ensure equal participation across groups while enabling them to maintain strict exclusion criteria. So while the current study chose the above cut points, future studies may choose to be more relaxed with their exclusion criteria, leading to even larger samples and participant conversion rates than were found in the current study.

## Conclusion

In sum, the current study implemented previous recommendations to collect a large sample of data from individuals who use substances from across the United States. It contributes to a growing body of evidence showing that internet-based data collection can be accomplished quickly and can produce high-quality datasets if appropriate precautions and cleaning methods are followed. The study also seeks to encourage further research using online data collection methods, accounting for the limitations and expanding the methods by which researchers can ensure high-quality data collection.

## Supporting information

S1 Table Measures used in screening and cannabis and HRI studies. (DOCX)

S1 Fig Example advertisement. (TIF)

S2 Fig Alternate example advertisement. (TIF)

S3 Fig Splash page. (TIFF)

S2 Table Demographics of removed compared to retained participants. (DOCX)
