# Supplementary material for: A national study of substance use: Demonstrated use of recommendations for best practice online data collection
Source: PLoS One. 2025 Nov 10;20(11):e0336612. doi: 10.1371/journal.pone.0336612 (PMC12599918; doi:10.1371/journal.pone.0336612)
Supplement: S1 Table — (DOCX) [file pone.0336612.s001.docx]

| **Supplemental Table 1** | | | | |
| --- | --- | --- | --- | --- |
| **Measures used in screening and cannabis and HRI studies** | | | | |
| Measure Name | Brief Description of Information Collected | # of Questions | # of Subscales | Internal Consistency |
| **Screener Survey** |  |  |  |  |
| Demographic Information | Age, gender, race/ethnicity, marital status, sexuality, state of residence, urban zone, education, employment status, number of children in the home, income, political affiliation | 13 | - | - |
| Generalized Anxiety Disorder 7-item (GAD-7)^1^ | Symptoms associated with generalized anxiety disorder | 7 | 1 | α=0.92 |
| Center for Epidemiologic Studies Depression Scale, Revised (CESD)^2^ | Symptoms associated with depression in non-clinical populations | 20 | 1 | α=0.93 |
| Pain, Enjoyment of Life, and General Activity Scale (PEG)^3^ | Pain | 3 | 3 | α=0.73 – 0.89 |
| Mental Health | Engagement in mental health services in past 90 days | 1 | - | - |
| Substance Use | Substances used ever and in past 90 days | 2 | - | - |
| Alcohol Use Disorders Identification Test – Consumption Questions (AUDIT-C)^4,5^ | Alcohol use in the past year, presence of hazardous drinking and disordered use | 3 | 1 |  |
| Nicotine Use | Type and frequency of use of nicotine products in past 90 days | 1-12 | 1 | - |
| Texas Christian University Drug Screen - 5 (TCUDS-5)^6^ | Symptoms and problems related to substance use, presence of a substance use disorder | 14 | 1 | - |
| Perceived harm or benefit of substances | Perceived harm and benefit of substances, ratings from 1(least) - 10 (most) | 2 | 2 | - |
| **Cannabis-Specific Measures** |  |  |  |  |
| Type of cannabis consumed | Type of cannabis consumed in past 90 days and past 30 days (e.g., Δ - 9 THC, Δ - 8 THC, CBD, etc.) | 2 | - | - |
| Manner of cannabis consumption | Manner of consumption of cannabis (e.g., smoking dried flower by itself, smoking dried flower with tobacco; Eating edibles, etc.) | 2-16 | - | - |
| Amount of cannabis consumed | Estimated frequency of use and amount of type of product consumed per month in g or mg | 15 | - | - |
| Where cannabis was purchased | Where the cannabis was purchased (e.g., from a legal recreational dispensary) and where most purchased | 2 | - | - |
| Marijuana Effect Expectancy Questionnaire – Brief Version (MEEQ-B)^7^ | Expected effects of consumption of cannabis | 6 | 2 | α=0.42-0.60 |
| Comprehensive Marijuana Motives Questionnaire (CMMQ)^8–10^ | Motivation/reasons for cannabis use | 39 | 13 | Subscales α=0.78-0.95 |
| Posttraumatic Stress Disorder Checklist for DSM-5 (PCL-5)^11^ | Presence and problems associated with symptoms of PTSD | 20 | 1 | α=0.94 |
| Pittsburgh Sleep Quality Inventory (PSQI)^12,13^ | Self-perceived sleep quality | 19 | 7 | α=0.94 |
| Prodromal Questionnaire – Brief Version (PQ-B)^14,15^ | Presence and concern associated with positive psychotic symptoms | 42 | 1 | α=0.85 |
| Brief COPE^16,17^ | Coping and behavioral self-regulation | 28 | 14 | Subscales α=0.50-0.82 |
| **HRI-Specific Measures** |  |  |  |  |
| Substance use and treatment questionnaire | Substances used in past 365 days and past 30 days, heaviest frequency of use in their lifetime, overdose experiences and number experienced in lifetime, etc. | 22 | - | - |
| Reasons for using substances | Reasons for using substances, selecting all that apply and ranking from number one reason respondent uses substances to last or least applicable reason (e.g., enjoying the feeling, curiosity, easy availability, etc.) | 2 | - | - |
| Perceptions of One's Substance Use | Perceived seriousness of SU issues (not at all to extremely serious), how bothered respondent is by their SU (not at all to extremely bothered), and self-efficacy not to use (not at all confident to extremely confident) | 2 | - | - |
| Treatment Interest & Barriers | Perceived importance of receiving SU treatment (not at all to extremely important), current interest in receiving treatment, treatment settings/medications of interest, barriers to treatment enrollment (select all that apply and rank order), reasons for disinterest in enrolling in treatment | 2 | - | - |
| Internalized Stigma of Substance Use-9 (ISSU-9)^18,19^ | Adapted version of the Internalized Stigma of Mental Illness-9 examining effects of stigma related to SU on self-esteem and distress | 9 | - | - |
| Harm Reduction Intervention Perceptions Questionnaire (HRIPQ) | Access to, experience with, perceptions (effectiveness, beneficence, and harm) of, and likelihood of future use of HRIs | 54 | - | - |

**References**

1. Spitzer RL, Kroenke K, Williams JBW, Löwe B. A brief measure for assessing generalized anxiety disorder: the GAD-7. *Arch Intern Med*. 2006;166(10):1092-1097. doi:10.1001/archinte.166.10.1092

2. Eaton WW, Smith C, Ybarra M, Muntaner C, Tien A. Center for Epidemiologic Studies Depression Scale: Review and Revision (CESD and CESD-R). In: *The Use of Psychological Testing for Treatment Planning and Outcomes Assessment: Instruments for Adults, Volume 3, 3rd Ed*. Lawrence Erlbaum Associates Publishers; 2004:363-377.

3. Krebs EE, Lorenz KA, Bair MJ, et al. Development and Initial Validation of the PEG, a Three-item Scale Assessing Pain Intensity and Interference. *J Gen Intern Med*. 2009;24(6):733-738. doi:10.1007/s11606-009-0981-1

4. Bush K, Kivlahan DR, McDonell MB, Fihn SD, Bradley KA. The AUDIT alcohol consumption questions (AUDIT-C): an effective brief screening test for problem drinking. Ambulatory Care Quality Improvement Project (ACQUIP). Alcohol Use Disorders Identification Test. *Arch Intern Med*. 1998;158(16):1789-1795. doi:10.1001/archinte.158.16.1789

5. Bradley KA, DeBenedetti AF, Volk RJ, Williams EC, Frank D, Kivlahan DR. AUDIT-C as a Brief Screen for Alcohol Misuse in Primary Care. *Alcoholism: Clinical and Experimental Research*. 2007;31(7):1208-1217. doi:10.1111/j.1530-0277.2007.00403.x

6. Knight DK, Blue TR, Flynn PM, Knight K. The TCU Drug Screen 5: Identifying Justice-involved Individuals with Substance Use Disorders. *J Offender Rehabil*. 2018;57(8):525-537. doi:10.1080/10509674.2018.1549180

7. Torrealday O, Stein LAR, Barnett N, et al. Validation of the Marijuana Effect Expectancy Questionnaire-Brief. *Journal of Child & Adolescent Substance Abuse*. 2008;17(4):1-17. doi:10.1080/15470650802231861

8. Lee CM, Neighbors C, Hendershot CS, Grossbard JR. Development and Preliminary Validation of a Comprehensive Marijuana Motives Questionnaire. *J Stud Alcohol Drugs*. 2009;70(2):279-287.

9. Bohnert KM, Bonar EE, Arnedt JT, Conroy DA, Walton MA, Ilgen MA. Utility of the comprehensive marijuana motives questionnaire among medical cannabis patients. *Addictive Behaviors*. 2018;76:139-144. doi:10.1016/j.addbeh.2017.08.001

10. Fales JL, Ladd BO, Magnan RE. Pain Relief as a Motivation for Cannabis Use Among Young Adult Users With and Without Chronic Pain. *The Journal of Pain*. 2019;20(8):908-916. doi:10.1016/j.jpain.2019.02.001

11. Blevins CA, Weathers FW, Davis MT, Witte TK, Domino JL. The Posttraumatic Stress Disorder Checklist for DSM-5 (PCL-5): Development and Initial Psychometric Evaluation. *Journal of Traumatic Stress*. 2015;28(6):489-498. doi:10.1002/jts.22059

12. Buysse DJ, Reynolds CF, Monk TH, Berman SR, Kupfer DJ. The Pittsburgh sleep quality index: A new instrument for psychiatric practice and research. *Psychiatry Research*. 1989;28(2):193-213. doi:10.1016/0165-1781(89)90047-4

13. Dietch JR, Taylor DJ, Sethi K, Kelly K, Bramoweth AD, Roane BM. Psychometric Evaluation of the PSQI in U.S. College Students. *Journal of Clinical Sleep Medicine*. 12(08):1121-1129. doi:10.5664/jcsm.6050

14. Loewy RL, Bearden CE, Johnson JK, Raine A, Cannon TD. The prodromal questionnaire (PQ): Preliminary validation of a self-report screening measure for prodromal and psychotic syndromes. *Schizophrenia Research*. 2005;79(1):117-125. doi:10.1016/j.schres.2005.03.007

15. Loewy RL, Pearson R, Vinogradov S, Bearden CE, Cannon TD. Psychosis risk screening with the Prodromal Questionnaire — Brief Version (PQ-B). *Schizophrenia Research*. 2011;129(1):42-46. doi:10.1016/j.schres.2011.03.029

16. Carver CS, Scheier MF, Weintraub JK. Assessing coping strategies: A theoretically based approach. *Journal of Personality and Social Psychology*. 1989;56(2):267-283. doi:10.1037/0022-3514.56.2.267

17. Carver CS. You want to measure coping but your protocol’ too long: Consider the brief cope. *Int J Behav Med*. 1997;4(1):92-100. doi:10.1207/s15327558ijbm0401_6

18. Boyd Ritsher J, Otilingam PG, Grajales M. Internalized stigma of mental illness: psychometric properties of a new measure. *Psychiatry Research*. 2003;121(1):31-49. doi:10.1016/j.psychres.2003.08.008

19. Boyd JE, Otilingam PG, DeForge BR. Brief version of the Internalized Stigma of Mental Illness (ISMI) scale: Psychometric properties and relationship to depression, self esteem, recovery orientation, empowerment, and perceived devaluation and discrimination. *Psychiatric Rehabilitation Journal*. 2014;37(1):17-23. doi:10.1037/prj0000035
