# Supplementary figures and images for: A national study of substance use: Demonstrated use of recommendations for best practice online data collection
Source: PLoS One. 2025 Nov 10;20(11):e0336612. doi: 10.1371/journal.pone.0336612 (PMC12599918; doi:10.1371/journal.pone.0336612)

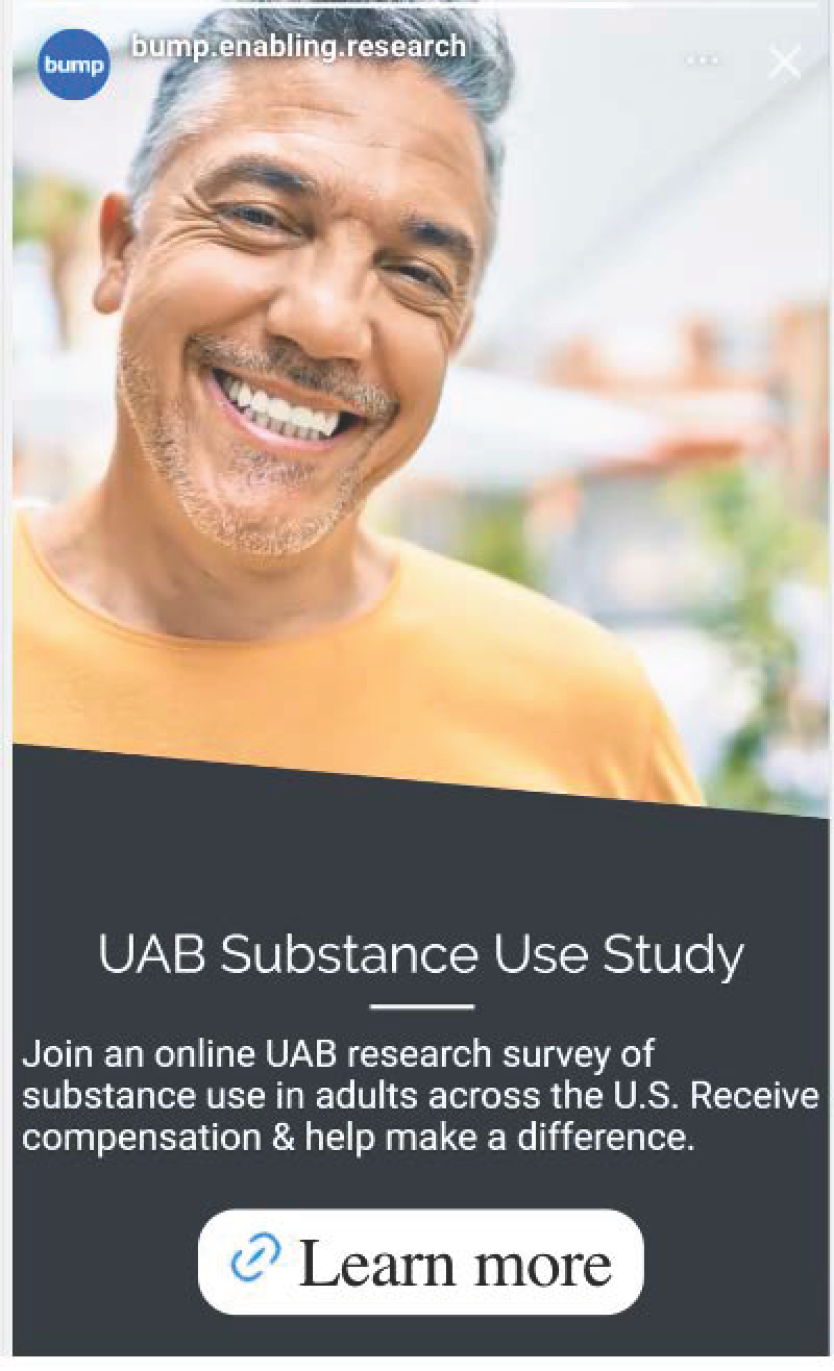

Supplement: S1 Fig — (TIF) [file pone.0336612.s002.tif]

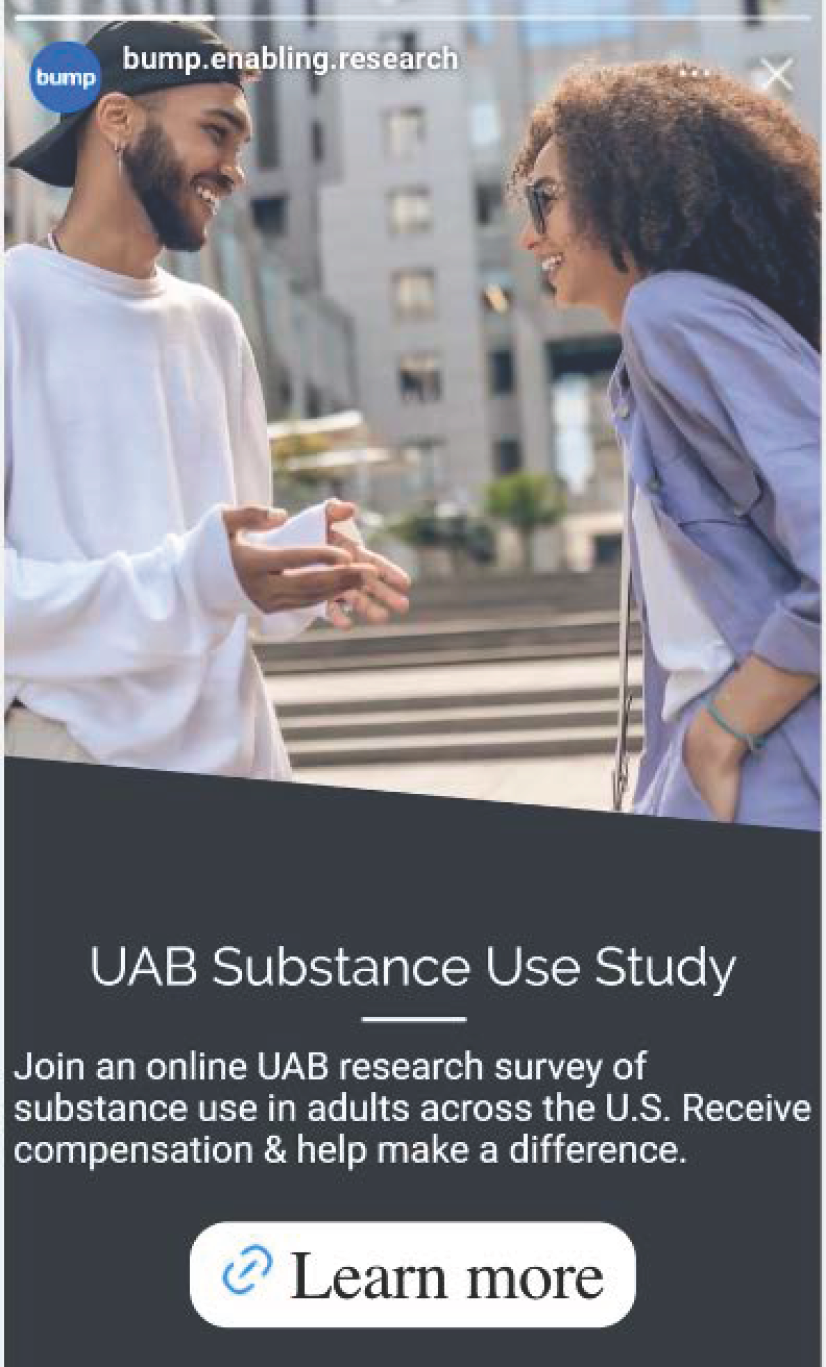

Supplement: S2 Fig — (TIF) [file pone.0336612.s003.tif]

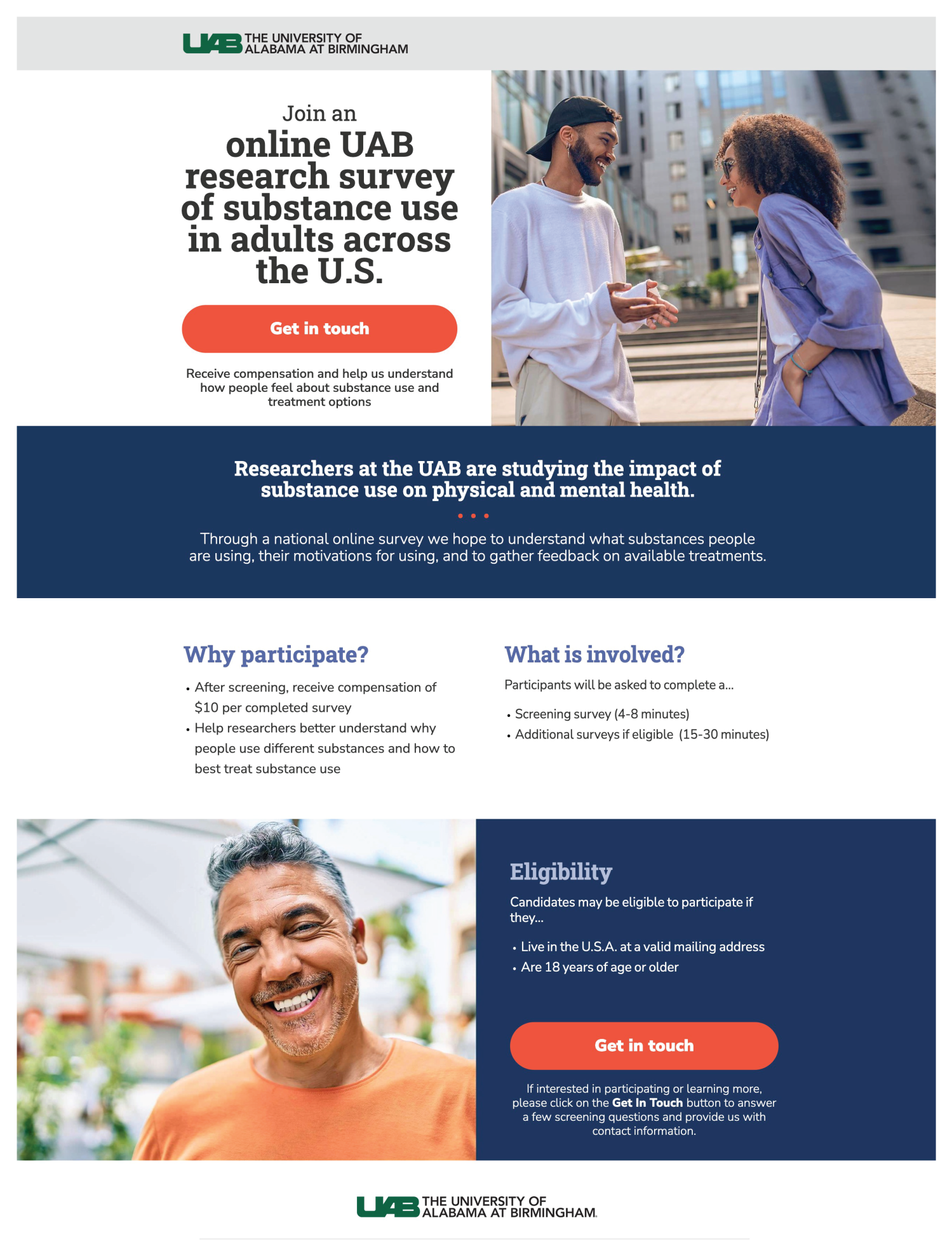

Supplement: S3 Fig — (TIFF) [file pone.0336612.s004.tiff]
