# Supplementary material for: A national study of substance use: Demonstrated use of recommendations for best practice online data collection
Source: PLoS One. 2025 Nov 10;20(11):e0336612. doi: 10.1371/journal.pone.0336612 (PMC12599918; doi:10.1371/journal.pone.0336612)
Supplement: S2 Table — (DOCX) [file pone.0336612.s005.docx]

| **S2 Table. Demographics of participants that were included vs. removed** | | | | |
| --- | --- | --- | --- | --- |
|  | Completed Cannabis Study | | Completed HRI Study | |
|  | Included | Removed | Included | Removed |
|  |  |  |  |  |
| **Total *N*** | 639 | 420 | 1137 | 433 |
|  | M | | | |
| **Age** | 43.58 | 42.13 | 45.41 | 44.4 |
|  | N (% of total) | | | |
| **Gender** |  |  |  |  |
| Male | 117 (27.7) | 180 (42.9) | 332 (29.2) | 153 (35.3) |
| Female | 441 (69.0) | 234 (55.7) | 785 (69.0) | 275 (63.5) |
| Non-binary | 9 (1.4) | 3 (0.7) | 12 (1.1) | 3 (0.7) |
| Trans Male | 5 (0.8) | 3 (0.7) | 5 (0.4) | 1 (0.2) |
| Trans Female | 4 (0.6) | 0 (0) | 1 (0.1) | 1 (0.2) |
| Other | 3 (0.5) | 0 (0) | 2 (0.2) | 0 (0) |
| **Race** |  |  |  |  |
| African-American/Black | 80 (12.5) | 88 (21.0) | 138 (12.1) | 69 (15.9) |
| Latino | 30 (4.7) | 17 (4.0) | 57 (5.0) | 20 (4.6) |
| Asian | 5 (0.8) | 4 (1.0) | 18 (1.6) | 3 (0.7) |
| Native American/Alaska Native | 5 (0.8) | 4 (1.0) | 11 (1.0) | 8 (1.8) |
| Caucasian/White | 458 (71.7) | 269 (64.0) | 806 (70.9) | 302 (69.7) |
| Arabic/Middle Eastern | 2 (0.3) | 3 (0.7) | 2 (0.2) | 1 (0.2) |
| Indian | 2 (0.3) | 1 (0.2) | 1 (0.1) | 1 (0.2) |
| Multiracial | 54 (8.5) | 31 (7.4) | 99 (8.7) | 28 (6.5) |
| Other Race | 3 (0.5) | 3 (0.7) | 5 (0.4) | 1 (0.2) |
| **Ethnicity** |  |  |  |  |
| Hispanic | 58 (9.1) | 42 (10.0) | 139 (12.2) | 45 (10.4) |
| Non-Hispanic | 581 (90.9) | 378 (90.0) | 998 (87.8) | 388 (89.6) |
